# Clinical Effectiveness and Cost-Effectiveness of Collaborative Treatment With Korean and Western Medicine for Primary Headache Disorders: Protocol for a Multicenter Prospective Observational Study

**DOI:** 10.2196/82819

**Published:** 2025-12-30

**Authors:** Jaeseung Kim, Jihwan Yun, Linae Kim, Shiva Raj Acharya, Changyon Han, NamKwen Kim

**Affiliations:** 1 College of Korean Medicine, Pusan National University Yangsan Republic of Korea; 2 Research Institute for Korean Medicine, Pusan National University Yangsan Republic of Korea; 3 Monitoring Center for Korean Medicine and Western Medicine Collaboration Yangsan Republic of Korea; 4 College of Medicine, Yonsei University Seoul Republic of Korea; 5 Graduate School of Public Administration, Seoul National University Seoul Republic of Korea

**Keywords:** primary headache disorders, collaborative treatment, cost-effectiveness, quality-adjusted life year, QALY

## Abstract

**Background:**

Primary headache disorders, including migraine and tension-type headache, are among the most prevalent neurological conditions worldwide, significantly contributing to disability and socioeconomic burden. While Western medicine (WM) predominantly focuses on pharmacological symptom management, Korean medicine (KM) emphasizes a holistic, individualized approach using modalities such as acupuncture and herbal medicine. Collaborative treatment, which combines these approaches, has been proposed as an alternative approach; however, robust evidence on its clinical effectiveness and cost-effectiveness remains limited.

**Objective:**

This study aims to evaluate the clinical effectiveness and economic value of collaborative treatment compared with usual care (UC) in patients with primary headache disorders in real-world clinical settings.

**Methods:**

This prospective, 2-arm, multicenter observational study will assess and compare the clinical effectiveness and economic value of collaborative treatment versus UC alone for patients with primary headache disorders. Adults aged ≥19 years with a primary diagnosis of primary headache disorders visiting participating hospitals under South Korea’s national collaborative treatment pilot project will be enrolled. Participants will receive either collaborative treatment (integrating WM pharmacotherapy with KM therapies such as acupuncture, pharmacopuncture, and herbal medicine) or UC (monodisciplinary care with WM or KM alone) based on informed choice. Clinical and cost-related outcomes will be assessed at baseline and 6 and 12 weeks. Clinical outcomes include monthly headache days, the numeric rating scale, the Headache Impact Test, the EQ-5D-5L, and the EuroQol visual analogue scale. The cost-effectiveness evaluation includes the cost per quality-adjusted life year, the incremental cost-effectiveness ratio, and the cost-effectiveness acceptability curve. Both intention-to-treat and per-protocol analyses will be performed.

**Results:**

The study protocol was approved by the institutional review boards in July 2025. The study is funded by the Ministry of Health and Welfare, Republic of Korea (grant 202500900001). Participant recruitment commenced in August 2025, aiming to enroll 312 patients across multiple centers. Data collection is currently ongoing and is projected to be completed by December 2027, with results expected to be published in 2028.

**Conclusions:**

This study is designed to generate real-world evidence on whether collaborative treatment yields superior clinical outcomes and greater cost-effectiveness compared to UC for primary headache disorders. The findings are expected to address existing evidence gaps by integrating multidimensional clinical and economic measures, supporting informed decision-making for health policy, and contributing to the advancement of collaborative treatment models for headache management within South Korea’s medical system and beyond.

**International Registered Report Identifier (IRRID):**

DERR1-10.2196/82819

## Introduction

Headache disorders are among the most prevalent neurological conditions worldwide, affecting over 52% of the global population and contributing substantially to disability and health care costs [1,2]. According to the Global Burden of Disease 2019 study, headache disorders rank as the leading cause of years lived with disability worldwide [1,3]. In South Korea, National Health Insurance Service (NHIS) data from 2023 indicate that 580,292 patients were treated for migraine and 543,750 were treated for other headache disorders, such as tension-type headache (TTH), with total health care expenditures exceeding KRW 255.82 billion (US $172.6 million) [4]. Additionally, chronic headaches can lead to reduced productivity, depression, and insomnia [5,6].

Headache disorders are classified into primary and secondary types; primary headache disorders, encompassing migraine and TTH, are estimated to account for approximately 90% of all headache cases [7]. Primary headache disorders are typically managed with pharmacological interventions, whereas the priority for secondary headache disorders lies in identifying and treating the underlying cause [8]. Pharmacological treatments for primary headache disorders—such as nonsteroidal anti-inflammatory drugs and tricyclic antidepressants—often demonstrate limited long-term efficacy and may cause adverse effects [9-11]. Chronic headache in particular is frequently resistant to pharmacotherapy, and overuse of analgesics can exacerbate headache symptoms [9,12].

These substantial impacts underscore the urgent need for structured, multidisciplinary national strategies and alternative approaches for the management and treatment of primary headache disorders [5,13].

Korean medicine (KM) uses a whole person–centered approach, identifying health conditions not merely as localized pathologies but as “patterns” that reflect the systemic active response of the patient. Unlike a disorder diagnosis, which focuses on the pathological process itself, pattern differentiation in KM incorporates systemic findings, innate or acquired constitutional traits, and the balance of yin and yang to capture the individual characteristics of the patient [14,15]. Specifically, electroacupuncture has proven effective in mitigating chronic headache severity, offering a viable option when pharmacological treatments yield limited results [16]. Collaborative treatment aims to integrate these holistic, restorative benefits of KM with the rapid symptom relief provided by Western medicine (WM) [17-20]. There is emerging evidence suggesting that collaborative care can reduce headache frequency and intensity more effectively than monotherapy alone [19,21]. However, robust clinical data comparing the effectiveness and cost-efficiency of collaborative treatment for various health conditions, including primary headache disorders, remain limited [22-24]. Since 1951, South Korea has maintained a dual medical system that legally recognizes both WM and KM [25]. While this structure preserves patient choice and cultural tradition, it can also result in overlapping resources and practice-related disputes [26]. To address these challenges, pilot programs were introduced to support WM-KM collaboration [27]. Despite these initiatives, barriers persist. Treatments from both disciplines provided on the same day for the same condition are typically not jointly reimbursed under the NHIS, resulting in higher out-of-pocket costs for patients [28]. Additionally, limited information exchange and the absence of standardized protocols hinder effective interdisciplinary coordination. To overcome these obstacles, the Korean government launched pilot programs that expanded insurance coverage for collaborative treatment, resulting in improved satisfaction among patients and medical professionals and reduced treatment duration [29,30].

Since its launch in 2016, South Korea’s pilot program for collaborative treatment has progressed through 4 stages and is now preparing for its fifth phase [28,30]. The program has gradually evolved from parallel practice to protocol-based models for specific conditions, along with a unified reimbursement system and strengthened institutional support for collaborative treatment [24,30,31]. Notable advancements include the introduction of interinstitutional electronic medical record sharing systems and joint training programs for WM and KM practitioners. Nonetheless, persistent challenges—such as fragmented delivery systems, insufficient clinical experience with interdisciplinary practice, and the lack of unified clinical guidelines—continue to limit the wider adoption of collaborative care models. To address these gaps and validate the feasibility of a structured collaborative model before a large-scale trial, preliminary data are required. Before designing this multicenter trial, we conducted an internal pilot study (KCT0010198) involving 59 patients with primary headache disorders to assess feasibility and preliminary efficacy.

The primary objective of this multicenter study is to investigate whether collaborative treatment provides greater clinical effectiveness and cost-efficiency for patients with primary headache disorders compared to usual care (UC) alone. We hypothesize that patients receiving collaborative treatment will exhibit significantly greater improvements in headache intensity (numeric rating scale; NRS) and frequency (monthly headache days; MHDs) compared to those receiving UC and that collaborative treatment will prove to be a cost-effective strategy from a societal perspective. To achieve this, we will conduct a prospective, multicenter observational study using validated patient-centered instruments focusing on headache-related disability, quality of life, and self-rated health status alongside an economic evaluation based on health care costs and utility measures.

## Methods

### Study Design

**Figure 1:**
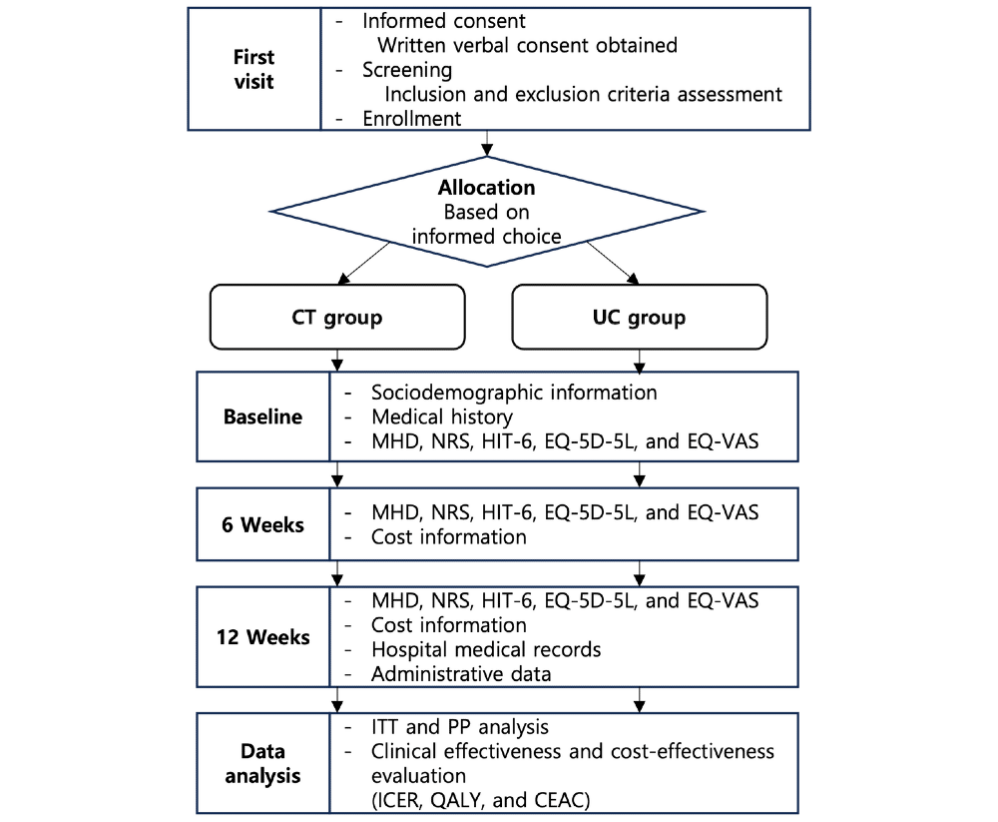
Schematic figure displaying the study flow and timing of assessments. CEAC: cost-effectiveness acceptability curve; CT: collaborative treatment; EQ-VAS: EuroQol visual analogue scale; HIT-6: Headache Impact Test; ICER: incremental cost-effectiveness ratio; ITT: intention to treat; MHD: monthly headache day; NRS: numerical rating scale; PP: per protocol; QALY: quality-adjusted life year; UC: usual care.

This is a prospective, 2-arm, multicenter observational study of patients with primary headache disorders at medical institutions participating in the fifth phase of the national pilot project for collaborative treatment [28]. The study protocol was developed following the SPIRIT (Standard Protocol Items: Recommendations for Interventional Trials) guidelines (Multimedia Appendix 1) and registered with the Clinical Research Information Service of South Korea (registration KCT0010812; July 28, 2025) [32]. The study will be conducted as part of the Registry for Korean Medicine and Western Medicine Collaborative Treatment study, with participant recruitment scheduled from the date of institutional review board approval to December 12, 2025. The detailed study schedule, as outlined in the SPIRIT guidelines, is illustrated in Table 1. Additionally, a schematic diagram displaying the study flow and timing of assessments is presented in Figure 1.

**Table 1:** Schedule of enrollment and assessments according to the SPIRIT (Standard Protocol Items: Recommendations for Interventional Trials) guidelines.

			Study stage						
			Enrollment		Baseline and allocation		Treatment and follow-up		
							6 weeks		12 weeks
**Enrollment**									
	Informed consent	✓							
	Inclusion and exclusion criteria	✓							
	Sociodemographic information and medical history			✓					
**Assessments**									
	MHDs^a^			✓		✓		✓	
	NRS^b^			✓		✓		✓	
	HIT-6^c^			✓		✓		✓	
	EQ-5D-5L			✓		✓		✓	
	EQ-VAS^d^			✓		✓		✓	
	Cost information					✓		✓	
	Hospital medical records and administrative data							✓	

^a^MHD: monthly headache day.

^b^NRS: numeric rating scale.

^c^HIT-6: Headache Impact Test.

^d^EQ-VAS: EuroQol visual analogue scale.

### Participants and Treatments

The study participants comprise patients diagnosed with primary headache disorders who visit hospitals participating in the fifth phase of the collaborative treatment pilot project and receive either collaborative treatment or UC alone [28]. Patients receiving UC are those treated exclusively with either KM or WM, whereas collaborative treatment refers to treatment involving both KM and WM. To minimize treatment heterogeneity and ensure clinical rigor, the therapeutic procedures for both WM and KM are conducted in strict accordance with the standard clinical practice guidelines (CPGs) for headaches in South Korea [10,33,34]. Specifically, following these standard CPGs, WM interventions typically involve acute abortive medications (eg, nonsteroidal anti-inflammatory drugs and triptans) and prophylactic agents (eg, β-blockers, anticonvulsants, and antidepressants) tailored to the specific headache disorder. Similarly, KM interventions are standardized to include acupuncture, electroacupuncture, pharmacopuncture (herbal injection), chuna manual therapy, and herbal prescriptions as outlined in the corresponding CPGs. In the collaborative treatment group, these standardized modalities are integrated to maximize therapeutic synergy; for instance, a patient may receive WM pharmacotherapy for rapid pain relief concurrently with KM acupuncture or chuna therapy to relieve muscle tension and address functional imbalances. Conversely, the UC group receives monodisciplinary care consisting exclusively of either WM or KM modalities. To ensure consistency across multiple centers, all participating practitioners will undergo standardized training on the study protocol and clinical guidelines before study initiation. Furthermore, adherence to these treatment standards will be periodically monitored by the Monitoring Center for Korean Medicine and Western Medicine Collaboration (MCMC) throughout the study period.

A nonrandomized observational design was adopted to reflect real-world clinical settings where patient preference acts as a crucial determinant of treatment adherence and outcomes. Consequently, participants will exercise informed choice regarding their treatment modality. During the consultation, practitioners will provide comprehensive information on the expected benefits, potential side effects, and cost implications of both collaborative treatment and UC. On the basis of this shared decision-making process, patients will select their preferred treatment group, ensuring that the study findings mirror actual health care use patterns in South Korea. If a participant opts for collaborative treatment, one practitioner will initiate a collaborative treatment request to the other practitioner. Treatment choices can be adjusted at any time through consultation with practitioners, and all treatments will be recorded and monitored. Patients who agree to participate will be given detailed information about the study and all safety-related aspects before they provide informed consent. After obtaining written consent, a medical doctor or a KM doctor will assess the participant’s eligibility. Those who meet the inclusion criteria will be enrolled in the study and assigned an enrollment number to preserve anonymity. To ensure fair and open participation, announcements about the study will be made publicly available at the participating institutions.

### Inclusion Criteria

Inclusion criteria are as follows: (1) adults aged ≥19 years; (2) individuals with a primary diagnosis of primary headache disorders who are first-time outpatients at one of the participating institutions as systematically defined by the *International Classification of Diseases, 10th Revision* (*ICD-10*; G43 [migraine], G44.0 [cluster headaches and other trigeminal autonomic cephalgias], G44.2 [TTH], and G44.8 [other specified headache syndromes]); and (3) individuals who voluntarily agree to participate in the study or whose legal representatives provide both written and verbal informed consent.

### Exclusion Criteria

Exclusion criteria are as follows: (1) individuals covered by automobile insurance (ineligible for treatment under the collaborative treatment pilot project) [28], (2) individuals with difficulty understanding the research questionnaire, (3) individuals with difficulty adhering to the study schedule and follow-up assessments, (4) individuals deemed unsuitable for participation at the discretion of the investigator, and (5) individuals participating in the national pilot project for herbal medicine coverage under the NHIS [28].

### Sample Size Estimation

To determine the appropriate sample size, we referenced data from our preceding pilot study, which was a multicenter prospective observational study conducted across 5 medical institutions during the fourth phase of the national collaborative treatment pilot project (December 2023-December 2024). This pilot study aimed to assess the feasibility and preliminary clinical effectiveness of collaborative treatment versus UC in patients with migraine (*ICD-10* code G43) and other headache syndromes (*ICD-10* code G44; Clinical Research Information Service registration KCT0010198). On the basis of the analysis of 59 participants from this pilot study, the NRS values were derived (group 1: mean 1.85, SD 1.66; group 2: mean 2.48, SD 2.13). Using the G*Power program with a 2-sided significance level of .05 and power of 80% (α=.05) and adjusting for a 10% discontinuation rate, the total required sample size for the study was determined to be 312 participants.

### Data Collection and Management

Data for the study will be collected through participant surveys using a standardized case report form (CRF). The CRF is designed to capture participants’ baseline characteristics, hospital medical records, and administrative data from each participating institution. This includes sociodemographic information, medical history, clinical indicators, and cost-related details. Administrative and medical data will be used to assess treatment costs associated with primary headache disorders, types and frequencies of treatment, medical expenses, duration of health care use, and types of services used. Trained and experienced researchers at each institution will be responsible for collecting both baseline and follow-up data. Face-to-face or telephone interviews will be conducted. All data will be entered and managed using the Internet-Based Clinical Research and Trial Management System developed by the Korea National Institute of Health. To protect participant confidentiality, all collected documents will use identification codes instead of personal names and will be stored as encrypted files, with access limited to authorized researchers. The MCMC will independently oversee data handling, storage, and validation throughout the study. This center, established under the Ministry of Health and Welfare as part of the fifth phase of the national pilot project, will serve as the study’s executive data management body. The monitoring center personnel will ensure compliance with the observational study protocol, authorize any modifications to the registry protocol, oversee the accuracy and appropriateness of data collection, and verify that informed consent has been properly obtained from all participants.

### Ethical Considerations

The study adheres to the principles outlined in the Declaration of Helsinki and follows good research practices as recommended by the Professional Society for Health Economics and Outcomes Research [35]. This study protocol was reviewed and approved by the institutional review boards of Wonkwang University Gwangju Oriental Medicine Hospital (approval WKIRB-2025-10) and Dongshin University Mokpo Oriental Medicine Hospital (approval DSMOH25-4). Written informed consent will be obtained from all participants or their legal representatives before enrollment. Participants will be fully informed about the study’s purpose, procedures, and potential risks and their right to withdraw at any time without penalty. To ensure privacy and confidentiality, all participant data will be deidentified and assigned a unique study identification code. Personal information will be stored separately from clinical data in encrypted files accessible only to authorized research personnel. No financial compensation is provided for participation.

### Measures

#### Baseline Characteristics

The participants’ baseline information will be collected on the first hospital visit. This includes age, gender, monthly household income (in KRW), history of treatment for primary headache disorders and other medical conditions, medication use, occupation and employment status, insurance status, and onset duration (duration from headache onset to first hospital visit).

#### Clinical Effectiveness

The clinical effectiveness of the treatment will be evaluated using MHDs [36], the NRS [37], the Headache Impact Test (HIT-6) [38,39], the EQ-5D-5L [40], and the EuroQol visual analogue scale (EQ-VAS) [41], with assessments conducted at each study time point: baseline and 6 and 12 weeks. Evidence indicates that increases in MHDs are linked to lower health-related quality of life (HRQOL) and that a reduction in MHDs by ≤4 contributes to improved patient health outcomes [36,42]. Thus, assessing MHDs in this study is crucial for evaluating treatment efficacy. The NRS measures overall pain intensity and discomfort associated with headache, with patients rating their pain on a scale from 0 (no pain) to 10 (worst pain imaginable) [37,43].

The HIT-6 is a widely used validated tool designed to measure the impact of headaches on a patient’s ability to function normally in daily life, comprising 6 items, each with 5 graded response options [38,39]. Each response is assigned a score ranging from 6 to 13 points per item, resulting in total scores that range from 36 to 78, with higher scores indicating greater impact or burden of headaches [44]. The patients’ HRQOL will be evaluated using the Korean version of the EQ-5D-5L [45]. The EQ-5D-5L assesses HRQOL across 5 dimensions: mobility, self-care, usual activities, pain and discomfort, and anxiety and depression [40,46]. The EQ-VAS will be used to measure the patients’ self-perceived health status on a scale from 0 to 100 [41,47]. Higher scores on the EQ-5D-5L and EQ-VAS indicate better HRQOL and overall health, respectively.

#### Cost-Effectiveness

Cost use for both treatments will be comprehensively assessed, including direct medical costs, direct nonmedical costs, and indirect costs such as productivity losses, derived from CRFs and hospital administrative data. The cost-effectiveness of the treatments will be evaluated using several measures: cost per quality-adjusted life year (QALY) [48,49], the incremental cost-effectiveness ratio (ICER) [50], and the cost-effectiveness acceptability curve (CEAC) [51]. The cost per QALY will be estimated using the area under the curve method [48,52] based on utility scores captured through the EQ-5D-5L and EQ-VAS. The ICER, representing the economic value of collaborative treatment compared with UC, is determined by dividing the incremental cost by the incremental QALYs gained [50,53]. The CEAC illustrates the probability that a treatment is cost-effective across a range of willingness-to-pay thresholds per QALY, with the horizontal axis denoting willingness-to-pay values and the vertical axis representing the corresponding probabilities [51,54].

### Safety

This study establishes standardized guidelines for reporting adverse events during the study period. All abnormal symptoms, adverse effects, or illnesses occurring in participants—regardless of their relation to the treatment received—will be documented. Recorded data will include a description of the event, time of onset, severity assessment by the practitioner, and relationship to the treatment. The research team will assess participant eligibility and determine the need for withdrawal. Additional visits may be scheduled for further evaluation. For participants who withdraw, the reasons will be documented, assessed, and followed up on accordingly.

### Statistical Analyses

Baseline data will be summarized using descriptive statistics such as percentages, frequencies, and means and SDs for both the UC and collaborative treatment groups. The UC group refers to participants receiving treatment exclusively with either KM or WM, whereas the collaborative treatment group includes those receiving both KM and WM. Data normality will be evaluated using the Shapiro-Wilk test and *Q*-*Q* plots. Between-group differences will be assessed using chi-square tests (Fisher exact test) for categorical data and the 2-tailed Student *t* test or ANOVA for continuous data. When normality or equal variance assumptions are violated, the Mann-Whitney *U* test and Kruskal-Wallis test will be performed.

Per-protocol and intention-to-treat (ITT) analyses will be conducted to assess clinical effectiveness and cost-effectiveness. To establish the ITT dataset, the proportion and mechanism of missing data will be evaluated using the Little test. If the data are identified as missing completely at random or missing at random, as defined by Rubin [55], multiple imputation will be performed [55,56]. Mean changes in clinical outcomes over time will be compared between groups as part of the effectiveness analysis. Furthermore, a generalized linear mixed model will be used to assess between-group effects at each time point. To rigorously control for heterogeneity and potential confounders, the model will adjust for fixed effects, including age, sex, headache history (onset duration), specific diagnosis, and comorbidities. While specific treatment modalities vary, reflecting real-world practice, the analysis adheres to the ITT principle to evaluate the effectiveness of the collaborative treatment strategy compared to UC rather than the efficacy of individual modalities. Subgroup analyses will be performed according to headache type (migraine, TTH, or other) and headache severity (episodic or chronic).

Utility values from the EQ-5D-5L and EQ-VAS will be used to estimate QALYs using the area under the curve method [48]. Deterministic analyses for the ICER will be performed using both ITT and per-protocol data from a limited societal perspective and a societal perspective. A probabilistic sensitivity analysis will also be carried out using parameter distributions and estimates. To account for sampling uncertainty in the ICER point estimates, 95% CIs will be calculated using the bootstrapping method or a seemingly unrelated regression model. All statistical analyses will be conducted using Stata/MP (version 14.0; StataCorp) and SAS (version 9.4; SAS Institute), with the significance level set at a *P* value of <.05.

## Results

The study was funded in June 2025. This trial protocol was approved by the institutional review boards in July 2025. Participant recruitment commenced in August 2025, with a target enrollment of 312 patients across multiple clinical centers. As of December 2025, participant recruitment is ongoing. Baseline data collection includes sociodemographic characteristics, clinical history, and treatment preferences. Clinical outcomes (MHDs, NRS, and HIT-6), HRQOL (EQ-5D-5L and EQ-VAS), and economic measures (QALYs, ICER, and CEAC) are being systematically collected during the study period. Data collection is expected to be completed by December 2027, and results are expected to be published in summer 2028.

## Discussion

### Anticipated Findings and Comparison to Prior Work

We hypothesize that collaborative treatment will provide greater reduction in headache frequency and intensity compared to UC while demonstrating its cost-effectiveness from a societal perspective. By incorporating real-world data from multiple medical institutions participating in the fifth phase of the national collaborative treatment pilot project, the study integrates multidimensional patient-centered measures to provide evidence supporting effective and sustainable collaborative treatment integration in headache treatment.

Previous studies on KM, WM, or collaborative treatment for headaches have shown potential benefits, including pain reduction and improved quality of life [10,18,19,22,57]. However, the evidence remains limited and inconsistent, often relying on single-center designs and short follow-up periods and lacking cost-effectiveness analyses, thereby reducing both generalizability and methodological rigor [17,18,58].

Unlike prior research, this study intends to address these gaps by using a multicenter prospective design with a comprehensive cost-effectiveness analysis, thereby aiming to provide more generalizable evidence on the clinical and economic value of integrating pharmacological and nonpharmacological interventions. Furthermore, this study follows the research framework previously applied in a study for facial palsy [24], supporting the feasibility of our approach.

### Strengths and Limitations

The strength of this study lies in its real-world, multicenter design within South Korea’s national health care framework, enhancing the generalizability of the findings. To our knowledge, this is the first study to comprehensively evaluate both the clinical effectiveness and cost-effectiveness of collaborative treatment for primary headache disorders. In interpreting the findings, certain methodological considerations inherent to this observational design should be noted. While the nonrandomized design and patient-driven treatment choices introduce potential selection bias, these features are inherent to observational studies reflecting actual clinical practice. To rigorously mitigate these factors, we will use generalized linear mixed models to control for baseline differences and injury mechanisms. Additionally, although the exclusion of patients covered by automobile insurance may limit generalizability to specific posttraumatic cases, this criterion aligns with the current operational guidelines of the national collaborative treatment pilot project. Regarding potential heterogeneity across institutions, standardized treatment protocols based on CPGs have been implemented across all centers to ensure consistency. Finally, cost data derived from patient self-reports will be cross-verified where possible to minimize recall bias. Finally, as this study is embedded within South Korea’s dual health care system, the findings may have specific relevance to similar integrative models but require careful adaptation for countries with different health care structures.

### Dissemination

The findings of this study will be disseminated through academic conferences and seminars and published in peer-reviewed journals. In addition, the results will inform the development of CPGs for the use of collaborative treatment for primary headache disorders. Progress updates will be reported annually, and ongoing information and additional resources will be made available through the MCMC’s dedicated website.

### Future Directions and Conclusions

If our hypotheses are confirmed, this study will generate robust, actionable evidence on the clinical effectiveness and cost-effectiveness of collaborative treatment for primary headache disorders. Beyond confirming efficacy, the findings will provide critical data to standardize collaborative treatment protocols and optimize insurance reimbursement models. This contributes to the growing body of evidence supporting collaborative approaches in chronic disease management, including primary headache disorders, and may help inform policy development for collaborative treatment–based health care delivery in South Korea and beyond.

## Appendix

Multimedia Appendix 1 SPIRIT 2025 checklist.

## Abbreviations

CEAC: cost-effectiveness acceptability curve
CPG: clinical practice guideline
CRF: case report form
EQ-VAS: EuroQol visual analogue scale
HIT-6: Headache Impact Test
HRQOL: health-related quality of life
ICD-10: International Classification of Diseases, 10th Revision
ICER: incremental cost-effectiveness ratio
ITT: intention to treat
KM: Korean medicine
MCMC: Monitoring Center for Korean Medicine and Western Medicine Collaboration
MHD: monthly headache day
NHIS: National Health Insurance Service
NRS: numeric rating scale
QALY: quality-adjusted life year
SPIRIT: Standard Protocol Items: Recommendations for Interventional Trials
TTH: tension-type headache
UC: usual care
WM: Western medicine

## Data Availability

Data sharing is not applicable to this paper as no datasets were generated or analyzed during this study.
